# An Evaluation of the Effectiveness of Ibuprofen and Manual Therapy in Young Women with Dysmenorrhea—A Pilot Study †

**DOI:** 10.3390/healthcare9060617

**Published:** 2021-05-21

**Authors:** Zofia Barcikowska, Elżbieta Rajkowska-Labon, Magdalena Emilia Grzybowska, Rita Hansdorfer-Korzon, Piotr Wąż, Katarzyna Zorena

**Affiliations:** 1 Department of Immunobiology and Environment Microbiology, Medical University of Gdańsk, Dębinki 7, 80-211 Gdańsk, Poland; kzorena@gumed.edu.pl; 2 Department of Physical Therapy, Medical University of Gdańsk, Dębinki 7, 80-211 Gdańsk, Poland; erlabon@gumed.edu.pl (E.R.-L.); rita.hansdorfer-korzon@gumed.edu.pl (R.H.-K.); 3 Department of Gynecology, Gynecologic Oncology and Gynecologic Endocrinology, Medical University of Gdańsk, Smoluchowskiego 17, 80-214 Gdańsk, Poland; mlgrzybowska@wp.pl; 4 Department of Nuclear Medicine, Medical University of Gdańsk, Tuwima 15, 80-210 Gdańsk, Poland; piotr.waz@gumed.edu.pl

**Keywords:** young women, dysmenorrhea, manual therapy, ibuprofen, progesterone, 17-beta estradiol, muscle tenderness and flexibility

## Abstract

The aim of the study was to evaluate the effect of manual therapy and the use of ibuprofen on the severity of dysmenorrhea and changes in the level of sex hormones in young women with dysmenorrhea. *Material and methods*: The study included six women, aged 22 ± 2 years, with primary dysmenorrhea (PD). A physiotherapist examined the tenderness and flexibility of the muscles. The patients were subjected to a gynecological and physiotherapeutic examination; the concentrations of progesterone and 17-beta-estradiol were also determined. In subgroup A (*n* = 3), manual therapy was performed 3 × 45 min; in subgroup B (*n* = 3), the patients received ibuprofen 3 × 400 mg/day. *Results*: In subgroup A, all patients showed a decrease in the level of progesterone and an increase in the concentration of estradiol. In subgroup B, the concentration of progesterone and 17-beta estradiol decreased in two subjects. In subgroup A, manual therapy reduced the severity of headache, back pain, diarrhea, fatigue, and PMS. In subgroup B, the use of ibuprofen only alleviated back pain and fatigue. Moreover, in subgroup A, after the application of manual therapy, improvement in flexibility and pain relief of the examined muscles was demonstrated. On the other hand, in subgroup B, no improvement in flexibility or reduction in muscle soreness was found in patients who took ibuprofen. *Conclusions*: Manual therapy may reduce menstrual pain in women with dysmenorrhea. However, the results need to be confirmed in studies conducted on a larger group of patients with dysmenorrhea.

## 1. Introduction

Previous research has demonstrated that dysmenorrhea is a complex process that depends on various factors [1,2,3]. Both our study and research conducted by other authors have shown that the following factors may have an impact on the development of dysmenorrhea: genetic causes, including the condition diagnosed in the mother and sisters, premenstrual syndrome (PMS), early age of menarche, stressful lifestyle, the lack of physical activity, loss of weight, unhealthy habits, social problems, depression, and even low self-esteem [4,5,6,7]. Other authors have demonstrated that hormones, particularly increased secretion of prostaglandins, may cause dysmenorrhea [3,4,5]. Prostaglandins lead to excessive contractile activity of the uterus, with the consequent hypoxia and pain [2]. In addition to prostaglandins, markers of inflammation and oxidative stress participate in the pathomechanism of dysmenorrhea [3,8].

The menstrual cycle is determined by cyclical changes in the level of hormones, whose secretion is regulated by the feedback system of the hypothalamic–pituitary–gonadal axis [9]. In the first phase of the menstrual cycle, the follicular phase, the secretion of estradiol dominates; this hormone plays an important role in endometrial proliferation and remodeling [10]. In the second part of the cycle, the luteal phase, progesterone secretion peaks. Progesterone is produced by the corpus luteum. If an ovum is not fertilized, the corpus luteum disappears and the hormone level goes down about 3 days before the beginning of menstrual bleeding [11]. A decrease in the level of progesterone is responsible for an inflammatory reaction leading to exfoliation of the endometrium and menstrual bleeding. Progesterone also has an impact on the regulation and synthesis of prostaglandins [11].

Pharmacological treatment, especially nonsteroidal anti-inflammatory drugs, is generally recommended to treat dysmenorrhea in young women [12,13]. Recently, more and more studies have been published on nonpharmacological methods of reducing menstrual pain, including TENS, heat, acupuncture, kinesio taping, acupressure, physical activity, and manual therapy [14,15,16,17,18,19]. Manual therapy has been applied since antiquity; the procedure is aimed at treating dysfunction of the joints and soft tissues by using manipulation and mobilization [20]. Manual therapy is safe, and the risk of its side-effects is lower than for pharmacotherapy [21,22]. In addition, manual therapy is effective, not only in patients with musculoskeletal problems, but also in those with visceral conditions, such as dysmenorrhea [23,24,25].

Proctor et al. [26] described the mechanisms used to reduce menstrual pain by manipulation of the spine. The first mechanism is based on the assumption that a reduction in mobility of the Th10-L2 segments, which are responsible for the sympathetic and parasympathetic innervation of the uterus, may disturb innervation of the blood vessels within the organ, with the consequent uterine narrowing and ischemia. Dysmenorrhea is also likely to be connected with pain referred from the musculoskeletal structures sharing the same nerve roots with the uterus [27]. Therefore, restoring the correct spine mobility through manipulations may improve nerve conduction and, thus, uterine blood supply [26]. Manipulations of the sacral spine can reduce tension within the broad ligament of the uterus and the uterine nerve roots, thus relieving menstrual pain [27].

It is known that pain, including dysmenorrhea, causes stimulation of the sympathetic nervous system [28]. The stimulation of the sympathetic nervous system results in motor and biochemical reactions. During the action of the stress factor, which is pain, the secretion of proteolytic enzymes (e.g., kinins, prostaglandins) occurs. Stimulation of the sympathetic nervous system by pain results in motor reactions, e.g., increased muscle tension in the affected area. This could lead to compression of venous, arterial, and nerve vessels, obstructing vascular flow or neurotransmission, which may consequently irritate pain (receptor) nociceptors. Activation of nociceptors in muscles and fascia follows mechanical injury or the accumulation of endogenous substances (e.g., bradykinin, prostaglandins). Myofascial pain is responsible for a change in muscle tension [29]. The use of manual techniques in relieving pain is based on a reflex therapy model. The aim of this therapy is to provoke reactions from the parasympathetic system. On the other hand, other authors have shown that, during manual therapy, the vagus nerve may be stimulated indirectly [30,31]. The vagus nerve comes out of the skull and its fibers even reach the ovarian plexus, which goes to the ovary, uterine broad ligament, and fallopian tube, and communicates with the uterine plexus [30,31]. So far, it has been shown that stimulation of the vagus nerve inhibits the production of tumor necrosis factor alpha (TNFα) and can be used to alleviate chronic inflammation in women with dysmenorrhea [32]. Moreover, activation of the parasympathetic nervous system leads to vasodilation of the pelvic organs, increasing oxygen perfusion and, thus, reducing pain caused by ischemia [33]. We formulated a hypothesis that manual therapy can relieve menstrual pain in young women and, therefore, it can be an alternative to treat dysmenorrhea. Thus, the aim of our study was to compare the effectiveness of manual therapy and the use of ibuprofen in young women with dysmenorrhea.

## 2. Materials and Methods

**Figure 1:**
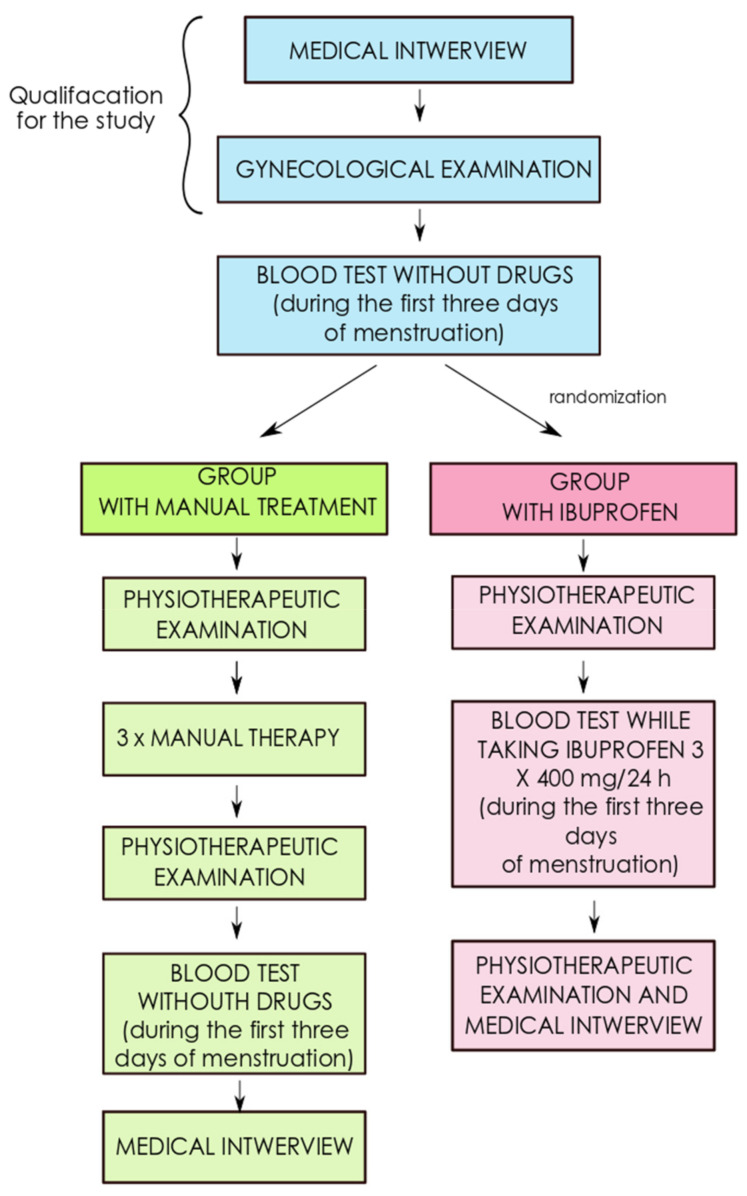
Participant flow diagram.

The study included six women, aged 22 ± 2 years, with dysmenorrhea. The subjects were recruited in 2019–2020 through verbal advertising, social media, and a gynecological outpatient clinic. The patients enrolled in the study were randomly divided into two equal subgroups A and B. Subgroup A included three women, aged 22 years, with dysmenorrhea. Three women, aged 21, were also qualified to subgroup B. The subjects from subgroup A underwent manual therapy, with three treatments of 45 min each. The patients from subgroup B received ibuprofen at a dose of 3 × 400 mg daily for the duration of menstrual pain (Figure 1). The participation in the study was voluntary and informed consent was provided. 

Qualification for the study was based on a structured interview. Women were asked about the regularity of the menstrual cycle, the duration of bleeding, the severity and duration of pain, the symptoms of premenstrual syndrome (PMS), and coexisting conditions (for instance, back pain, headache, vomiting, diarrhea, fainting, and fatigue). Patients were also asked about the reproductive system’s diseases, surgical procedures on the abdominal cavity and spine, medications taken regularly, and drugs used during menstruation. The study included nulliparous women in age 18-30 who had regular menstrual cycles, and who rated dysmenorrhea as five or more points on the NPRS [34]. The exclusion criteria were irregular menstrual cycle and irregular dysmenorrhea, diseases of the reproductive system (e.g., polycystic syndrome, endometriosis), taking hormonal contraception, contraindications for taking ibuprofen, hip dysplasia, Perthes disease, surgical interventions within the spine and abdominal region, and history of pelvic injuries, regular use of nonsteroidal anti-inflammatory drugs (NSAIDs), and secondary dysmenorrhea. The third part of the survey contained questions excluding from the study. The intensity of menstrual pain and the symptoms of PMS were evaluated using the 11-grade NPRS scale, where 0 indicates no pain and 10 indicates the most severe imaginable pain. The additional symptoms were assessed on a five-grade scale where “0” means none, “1” means mild, “2” means moderate, “3” means severe, and “4” means very severe [22]. Completion of the questionnaire was followed by a gynecological examination. A gynecologist performed a gynecological examination and a transvaginal ultrasound or transabdominal scan for women who had not begun sexual activity.

### 2.1. Testing the Concentration of Sex Hormones

After patients with dysmenorrhea were qualified for the study, 5 mL of blood was collected from each subject during the first 3 days of the menstrual cycle (between 7:30 and 9:45 a.m.) in order to evaluate the levels of progesterone and 17-beta-estradiol. Blood samples were analyzed according to the standard procedures of the Invicta Medical Diagnostic Laboratory in Gdańsk, Poland. The levels of 17-beta-estradiol and progesterone were determined using the ECLIA electrochemiluminescence method according to the manufacturer’s instructions. In each woman, blood tests were performed twice. In subgroup A, blood was collected before and after manual therapy; the patient could not take analgesics until blood was collected. In subgroup B, blood was collected first during menstruation without taking medications; then, during the next menstrual period, the blood was taken while taking ibuprofen.

### 2.2. Physiotherapeutic Examination

**Figure 2:**
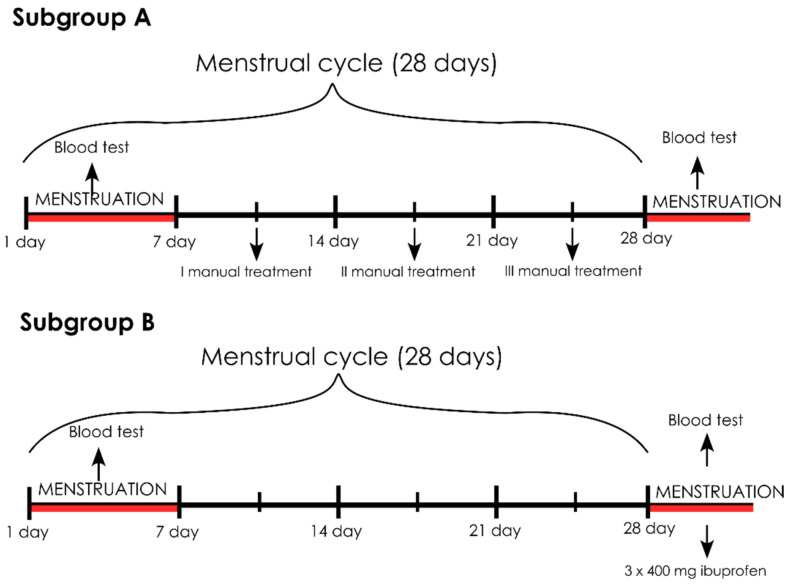
The scheme for carrying out the therapy in subgroups A and B.

After menstruation, women from subgroup A and subgroup B were subjected to a physiotherapeutic examination. A physiotherapist examined the soreness and flexibility of the following muscles: the diaphragm, iliopsoas muscles, adductors of the thighs, quadratus lumborum, hamstring group, piriformis muscles, and tensors fasciae latae. The tenderness of the muscles was examined by palpating the trigger points according to Simons and Travell (Figure 2) [35,36]. The tension and tenderness of the breathing diaphragm were examined while lying on the back with the legs bent, whereby a physiotherapist placed their hands on the ribs, from the cranial side, covering the inner part of the ribs with the fingertips. Breathing diaphragm flexibility was evaluated by measuring the chest circumference during maximum breathing in and breathing out. The measurements were taken using a tailor’s tape measure. The chest circumference was measured by placing the tap measure 8 cm below the xiphoid process of the sternum and 10 cm above the base of the sacrum; next, the differences between maximum inspiration and exhalation were calculated. Flexibility of the quadratus lumborum muscles was evaluated by performing a lateral bend while standing with the back against the wall. The subject made a lateral bend in both directions, moving her hand along the outer surface of the thigh. During bending, the upper anterior iliac spines were examined. Next, the distance was measured between the pulp of the third finger and the articular space of the knee joint. Flexibility of the iliopsoas was evaluated using the Thomas test [37]. Lying on her back, with the pelvis stabilized by the examiner, the patient pulled up the flexed lower limb opposite to that examined to the abdomen. The physiotherapist assessed whether the tested lower limb did not leave the ground. The modified Ober’s test was applied to assess the flexibility of tensors fasciae latae [38]. The test was carried out while lying on the untested side. The not examined lower limb was positioned in flexion of the hip and knee. The examiner held, with one hand, the tested lower limb in a neutral position of the hip joint, at the level of the knee, with the other hand placed at the level of the ankle. The patient was asked to relax her lower limb. The physiotherapist assessed whether the knee of the examined lower limb would fall on the couch. Lowering of the knee to the ground was considered the norm. The flexibility of the adductors of the thighs was evaluated by passive abduction of the lower limb. The flexibility of the hamstring group muscles was assessed by measuring the inferior complement angle using a goniometer at the knee joint. The examination was carried out in the supine position, with the hip and knee joints flexed to the angle of 90°. In this position, the examiner tried to passively straighten the lower leg. The movement was stopped when the resistance to the movement was too great. Then, the so-called the complement angle, i.e., the angle between the long axis of the lower leg and the vertical axis [39], was measured using the SAEHAN plastic universal two-arm goniometer with a 360° goniometer face and 30 cm (11.81 in) movable arms (SAEHAN Corp., Dangjin-gun, South Korea). All the examinations were carried out symmetrically on both sides. Additionally, in each patient, the position of the pelvis was evaluated, as well as pain and mobility of the hip and sacroiliac joints. In each patient, two physiotherapeutic examinations were performed—before and after the manual therapy.

### 2.3. Manual Therapy and Pharmacotherapy

**Figure 3:**
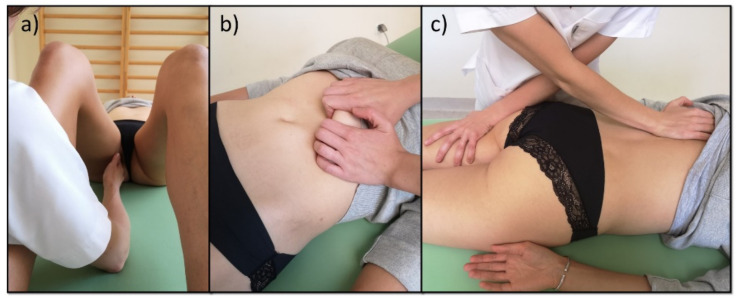
(**a**) Pelvic floor release; (**b**) stretching of breathing diaphragm; (**c**) release of the limbs with breathing diaphragm.

**Figure 4:**
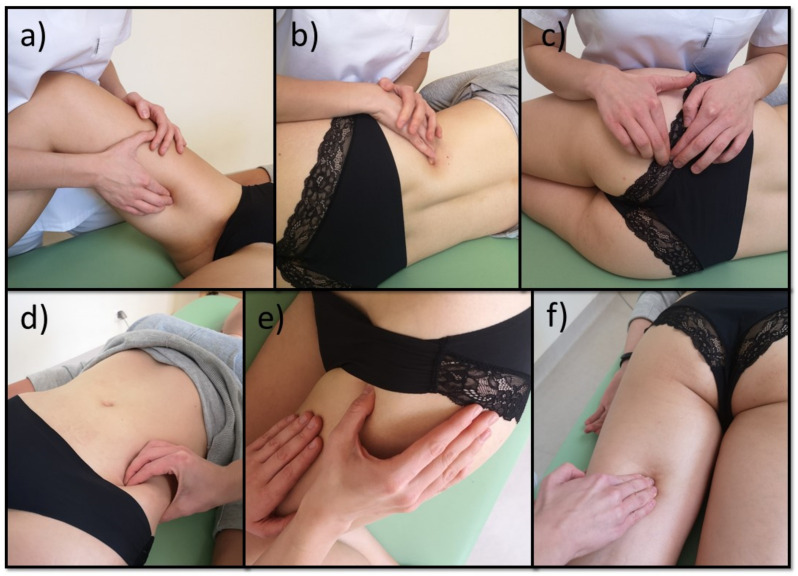
Therapy of tender points: (**a**) mm. adductors of the thigh; (**b**) mm. quadratus lumborum; (**c**) m. piriform; (**d**) m. iliopsoas; (**e**) m. tensor fasciae latae; (**f**) mm. hamstring.

**Figure 5:**
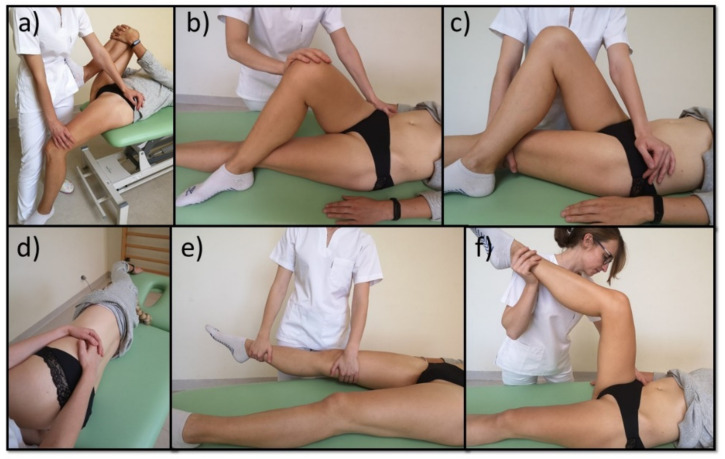
Post-isometric muscle release: (**a**) m. ilipsoas; (**b**) m. piriform; (**c**) m. m. tensor fasciae latae; (**d**) m. quadratus lumborum; (**e**) mm. adductors of the thigh; (**f**) mm. hamstring.

All women from subgroup A were subjected to manual therapy during one menstrual cycle. The therapy was performed once a week, and each session lasted 45 min. The subjects underwent three treatment sessions, depending on the duration of the menstrual cycle. The therapy included mobilization of the breathing diaphragm, post-isometric relaxation, and trigger point therapy according to Simons and Travell (Figure 3, Figure 4 and Figure 5) [35,36]. The therapy involved only those muscles that were dysfunctional on the physiotherapeutic examination. The tension of the pelvic floor muscles was normalized (Figure 3). After the entire manual therapy cycle, the physiotherapeutic examination was repeated in each patient. The blood test was repeated during the next menstrual cycle. Again, the subjects did not take any medication until blood was collected. After menstruation, the physiotherapist took the medical history once again in order to evaluate the effects of the therapy. The manual therapy and physiotherapeutic examination were performed by the same physiotherapist (Figure 2).

In subgroup B, there were no interventions during the menstrual cycle. During the next menstrual period, the patients were asked to take ibuprofen at a dose of 3 × 400 mg daily for the duration of pain (Figure 2).

The study was approved by the Bioethics Committee of the Medical University of Gdańsk (No. NKBBN/475/2018). The research was conducted in accordance with the principles of the Declaration of Helsinki as revised in 1996.

## 3. Results

### 3.1. The Characteristics of Patients with Dysmenorrhea from the Study Subgroup (A) and Control Subgroup (B)

Table 1 presents the characteristics of the subjects. In subgroup A (*n* = 3), the mean age of subjects was 22 years, body mass index (BMI) = 21 kg/m^2^, the mean duration of the menstrual cycle was 27 days, and the duration of menstruation was 5 days. In group B (*n* = 3), the mean age of patients was 21 years, BMI = 24 kg/m^2^, the mean duration of the menstrual cycle was 30 days, and the mean duration of menstruation was 6 days.

### 3.2. Patients with Dysmenorrhea Subjected to Manual Therapy

#### 3.2.1. Patient No. 1

A patient with dysmenorrhea, 24 years of age, with a BMI of 24 kg/m^2^. The physiotherapeutic examination showed the pelvis in anteversion.

The manual therapy led to an increase in the difference in chest circumferences measured during inhalation and exhalation from 7 to 11 cm. Pain and increased tension of the breathing diaphragm were reported during the first and second examination. Manual therapy reduced pain of the iliopsoas muscles, adductors of the thigh, tensors fasciae latae, piriformis, left quadratus lumborum, and hamstring group on the right side. Furthermore, an improvement in the flexibility of left tensor fasciae latae and quadratus lumborum muscles was observed. However, there were no changes in the flexibility of hamstring group muscles and right tensor fasciae latae.

**Figure 6:**
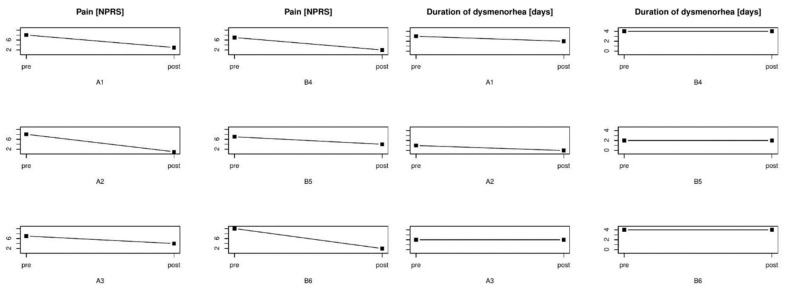
Intensity of pain in NPRS and duration of dysmenorrhea before and after manual therapy and taking ibuprofen. Abbreviations: A1, A2, A3, B1, B2, B3—patient designation, NPRS—Numeric Pain Rating Scale, Pre—result before treatment, Post—result after treatment.

After the manual therapy, the severity of menstrual pain decreased from eight to three points on the NPRS scale, with the duration of pain shortened from 3 to 2 days (Figure 6). Before manual therapy, the duration of the menstrual cycle was 28 days, while it was 30 days after the treatment. Both before and after the manual therapy, the menstruation lasted 6 days (Table 2). PMS scored eight points on the NPRS scale before the therapy vs. three points after the treatment. Before manual therapy, back pain was evaluated as four points, while it was two points after the treatment. Moreover, the feeling of fatigue before the therapy was rated as three, while it was rated as two after the treatment (Table 3).

Before the manual therapy, the level of progesterone was 0.54 ng/mL vs. 0.31 ng/mL after the treatment. The concentration of estradiol was 53 pg/mL and 60 pg/mL, before and after the treatment, respectively (Table 4).

#### 3.2.2. Patient No. 2

A patient with dysmenorrhea, 22 years of age, BMI 20 kg/m^2^. The pelvis was in an intermediate position.

The palpation examination performed before the manual therapy showed that the breathing diaphragm was overly tense and painful, with normal tension and no pain were reported after the treatment. The manual therapy led to a reduction in the difference in chest circumference during inhalation and exhalation (6 cm vs. 5 cm).

The manual therapy led to a reduction of pain of the quadratus lumborum muscles and the hamstring group on the right side. However, no changes were observed in the tenderness of the iliopsoas muscles, adductors, tensors fasciae latae, and piriformis muscles. The manual therapy did not improve the flexibility of the quadratus lumborum muscles, the hamstring group muscles, and tensors fasciae latae.

Before the therapy, the patient evaluated menstrual pain as eight points on the NPRS scale; after the treatment, the severity of menstrual pain decreased to one point, while the duration of menstrual pain was shortened by 1 day (Figure 6). The duration of the menstrual cycle before and after the treatment was 25 days, while the menstrual bleeding lasted for 5 days (Table 2). The patient showed reductions in the intensity of back pain from four to zero points, headache from three to zero points, fatigue from two to one point, and symptoms of PMS from five to two points (Table 3). Hormonal tests performed before and after the manual therapy revealed the levels of progesterone to be 0.42 ng/mL and 0.38 ng/mL, respectively. There was an increase in the concentration of estradiol, with 26 and 36 pg/mL recorded before and after the treatment (Table 4).

#### 3.2.3. Patient No. 3

A patient with dysmenorrhea, 20 years of age, BMI 20 kg/m^2^. The pelvis was set in anteversion. Before and after the therapy, the patient showed increased tension of the breathing diaphragm; the difference in chest circumference during inhalation and exhalation was 7 cm. After manual therapy, tenderness of the breathing diaphragm disappeared. Manual therapy reduced the soreness of the iliopsoas muscles, quadratus lumborum muscles, and piriformis muscles. Manual therapy did not affect the flexibility of the right tensor fasciae latae, quadratus lumborum muscles, and hamstring group muscles.

Before manual therapy, the patient scored her menstrual pain at seven points on the NPRS scale; after the treatment, the pain decreased by three points. The duration of pain, both before and after manual therapy, was 2 days (Figure 6). The duration of the menstrual cycle was 28 days and 33 days, respectively, whereas menstrual bleeding lasted 4 vs. 5 days (Table 2). After the manual therapy, the patient did not experience back pain or headache during menstruation (one and zero points), previously assessed at three points, while the severity of diarrhea decreased from three to one point. There was no change in the severity of vomiting and fatigue. After manual therapy, the patient did not experience the symptoms of PMS, which were initially rated at three points on the NPRS scale (Table 3).

After manual therapy, the patient showed a lower level of progesterone (0.26 ng/mL vs. 0.22 ng/mL) and a higher concentration of estradiol (5 pg/mL vs. 12 pg/mL) (Table 4).

### 3.3. Patients with Dysmenorrhea Treated with Ibuprofen

#### 3.3.1. Patient No. 4

A patient with dysmenorrhea, 22 years of age, BMI 24 kg/m^2^. The pelvis was set in retroversion. The examination performed before and after the administration of ibuprofen revealed pain and increased tension within the breathing diaphragm; the difference in chest circumference during inhalation and exhalation was 4 cm. The palpation examination showed no impact of ibuprofen on the reduction in pain within iliopsoas muscles, adductors of the thighs, quadratus lumborum muscles, piriformis muscles, and hamstring group muscles. There was also no influence on the flexibility of quadratus lumborum muscles, hamstring group muscles, and left tensor fasciae latae. During the second examination, the right tensor fasciae latae was sore, but its flexibility improved.

The patient evaluated menstrual pain without taking medications as seven points on the NPRS scale. While taking ibuprofen at a dose of 3 × 400 mg a day for the duration of pain, the pain decreased to two points on the NPRS scale (Figure 6). The menstrual cycle, both before and during the study, lasted 29 days, menstruation lasted 7 days, and menstrual pain lasted 4 days (Table 2). Initially, the patient did not have a headache, but pain occurred while taking ibuprofen and was scored as two points. No impact of the drug was observed on the severity of the diarrhea (four points) and fatigue (three points). The patient taking ibuprofen did not experience episodes of fainting (one vs. zero points) while taking the medication. However, during the observed menstrual cycle, the symptoms of PMS increased, and they were estimated at six points vs. seven points on the NPRS scale (Table 3).

The concentrations of hormones determined during the use of ibuprofen revealed a lower level of progesterone (0.58 vs. 0.33 ng/mL) and estradiol (13 pg/mL vs. <5 pg/mL) (Table 4).

#### 3.3.2. Patient No. 5

A patient with dysmenorrhea, 22 years of age, BMI 22 kg/m^2^. Upon physiotherapeutic examination, the pelvis was set in anteversion. The first physiotherapeutic examination showed no tenderness and normal tension of the breathing diaphragm; the difference in chest circumferences during inhalation and exhalation was 6 cm, while this difference was 5.5 cm during the second examination. The palpation examination revealed pain of the iliopsoas muscles, adductors of the thighs, and hamstring group muscles. There was shortening of the quadratus lumborum muscles and hamstring group muscles. During the second examination, a change was found in the right piriformis muscle because of its soreness. The patient was unable to evaluate the flexibility of the tensors fasciae latae due to her inability to relax the lower limb during the examination.

Initially, the patient scored menstrual pain at seven points, while it was scored as four points when taking ibuprofen on the NPRS scale (Figure 6). Before the study and during the follow-up, the menstrual cycle lasted 29 days, menstrual bleeding lasted 6 days, and menstrual pain lasted 2 days (Table 2). There was no influence of taking ibuprofen on the severity of back pain (two points) and fatigue (two points). A stronger headache of zero vs. two points was observed while taking ibuprofen. However, there was a decrease in the symptoms of PMS from three to zero points on the NPRS scale (Table 3).

Before pharmacological therapy (ibuprofen) was applied in the patient from subgroup B, the levels of progesterone were 0.21 ng/mL vs. 0.17 ng/mL after ibuprofen administration. On the other hand, the concentrations of estradiol before and after pharmacological therapy were 50 pg/mL and 21 pg/mL, respectively (Table 4).

#### 3.3.3. Patient No. 6

A patient with dysmenorrhea, 20 years of age, BMI 26 kg/m^2^. The pelvis was set in anteversion. There was no effect of taking ibuprofen on pain and increased tension of the breathing diaphragm. During the second examination, the difference in chest circumference on inhalation and exhalation decreased from 7.5 cm to 5 cm. Ibuprofen did not have an impact on the soreness of iliopsoas muscles, adductors, quadratus lumborum muscles, piriformis muscles, and tensors fasciae latae. Moreover, there were changes in the flexibility of the right iliopsoas muscle, adductors, quadratus lumborum muscles, and hamstring group muscles. In addition, the examination performed after the use of ibuprofen revealed soreness of the hamstring group muscles. The flexibility of tensors fasciae latae was not examined because the patient was unable to relax the lower limbs during the examination.

Ibuprofen reduced the severity of menstrual pain from 10 to two points on the NPRS scale; the menstrual pain lasted 4 days (Figure 6). The menstrual cycle lasted an average of 28 days; however, during the study, the menstrual cycle lasted 26 days, while menstrual bleeding lasted 6 days (Table 2). The administration of ibuprofen reduced back pain from three to two points, headache from two to zero points, vomiting from one to zero points, and fatigue from three to two points. The use of ibuprofen did not affect the severity of diarrhea (three vs. three points). On the other hand, the severity of PMS symptoms increased from seven to eight points on the NPRS scale (Table 3).

Before the pharmacological therapy (ibuprofen) was applied in the patient from subgroup B, the levels of progesterone were 0.14 ng/mL vs. 0.31 ng/mL after the ibuprofen administration. On the other hand, the concentrations of estradiol were 0.18 pg/mL and 25 pg/mL, respectively, before and after the pharmacological therapy (Table 4).

## 4. Discussion

Our physiotherapeutic examinations to evaluate the flexibility of muscles and the trigger points performed in all patients with dysmenorrhea showed disorders of the following muscles: iliopsoas, adductors of the thigh, quadratus lumborum muscles, and hamstring group muscles. Dysfunctions of the breathing diaphragm, piriformis muscles, and tensors fasciae latae were found in five subjects. In all patients with dysmenorrhea from subgroup A, manual therapy led to pain relief within the quadratus lumborum muscles. In two patients from subgroup A, manual therapy resulted in a reduction in pain within the iliopsoas, tensors fasciae latae, piriformis muscles, and hamstring group muscles, and it resulted in less severe pain of the adductors in one patient. In subgroup B, patients with dysmenorrhea receiving ibuprofen showed no improvement in flexibility and a reduction in muscle soreness. Moreover, one patient with dysmenorrhea from subgroup B showed excessive tension within the tensors fasciae latae and hamstring group muscles.

We believe that the decrease in soreness and improvement in muscle flexibility in patients from subgroup A resulted in a reduction in menstrual pain. An analgesic effect was obtained in all patients from subgroup A. The changes in the severity of menstrual pain ranged from three to eight points on the NPRS scale. As no studies have described dysmenorrhea therapy using post-isometric relaxation techniques and the therapy of trigger points, we refer our results to other forms of manual therapy, such as osteopathy and spine manipulation [27,40,41].The muscles subjected to therapy in our study had their attachments from the Th12 segment of the spine, through the lumbar segment to the sacrum and coccyx [42,43,44]. Parasympathetic (S3–S4) and sympathetic (L1–L3) innervation of the uterus also originates from this region. Thus, improvement in the flexibility of the muscles may lead to better nerve conduction, improved blood supply to the uterus, and a reduction in hypoxia.

The patients with dysmenorrhea from subgroup B who received ibuprofen also showed a reduction in the severity of menstrual pain by three to eight points on the NPRS scale. The comparable effectiveness of manual therapy and ibuprofen was also confirmed in the study conducted by Barassi et al., who used neuromuscular manual therapy and ibuprofen to reduce dysmenorrhea. The authors obtained a comparable analgesic effect to our results [45].

In addition to reducing menstrual pain, manual therapy shortened the duration of menstrual pain in two patients from subgroup A. This was not found in subjects from subgroup B while taking ibuprofen. The effect of manual therapy, i.e., shortening of the duration of menstrual pain, is consistent with the results obtained by Schwerla et al. [40], who demonstrated that menstrual pain in women after osteopathic therapy was shortened by 2 days [40].

Furthermore, in a further stage of our study, we attempted to assess the impact of manual therapy in patients from subgroup A and ibuprofen in subjects from subgroup B on the level of sex hormones.

In all the patients with menstrual pain from subgroup A, manual therapy resulted in a lower level of progesterone compared to the concentration before the treatment. In subgroup B, while taking ibuprofen, a lower concentration of progesterone was found in two patients. In patients with dysmenorrhea, manual therapy probably had an effect on the reduction in progesterone concentration. Our study results differ from the outcomes obtained by Roomruangwong et al., who showed that a lower level of progesterone is associated with a higher risk of menstrual pain and the related symptoms [46].

**Figure 7:**
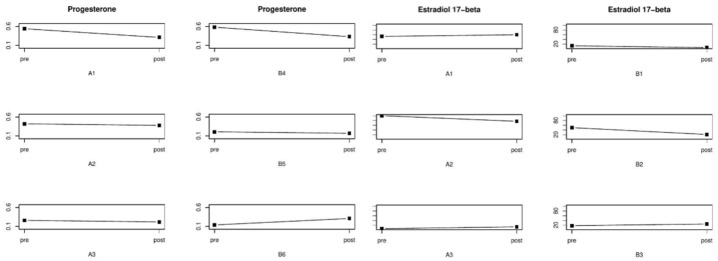
Concentration of progesterone and estradiol 17-beta in patients in subgroups A and B before and after treatment. Abbreviations: A1, A2, A3, B1, B2, B3—patient designation, NPRS—Numeric Pain Rating Scale, Pre—result before treatment, Post—result after treatment.

We analyzed the effect of manual therapy and the use of ibuprofen on the concentration of 17-beta-estradiol in young women with dysmenorrhea. Manual therapy led to a higher level of 17-beta-estradiol in all patients from subgroup A. In subgroup B, a lower concentration of 17-beta-estradiol was detected in two patients (Figure 7). There is no research into the effect of manual therapy on the level of 17-beta-estradiol. In one study, authors demonstrated the effect of acupuncture on the concentration of estradiol in perimenopausal women. Chinese researchers, who examined a group of perimenopausal women, checked whether acupuncture had an impact on hot flashes in menopause and the concentration of sex hormones, including estradiol. The authors found that the acupuncture therapy performed in perimenopausal women led to a higher concentration of estradiol [47].

**Figure 8:**
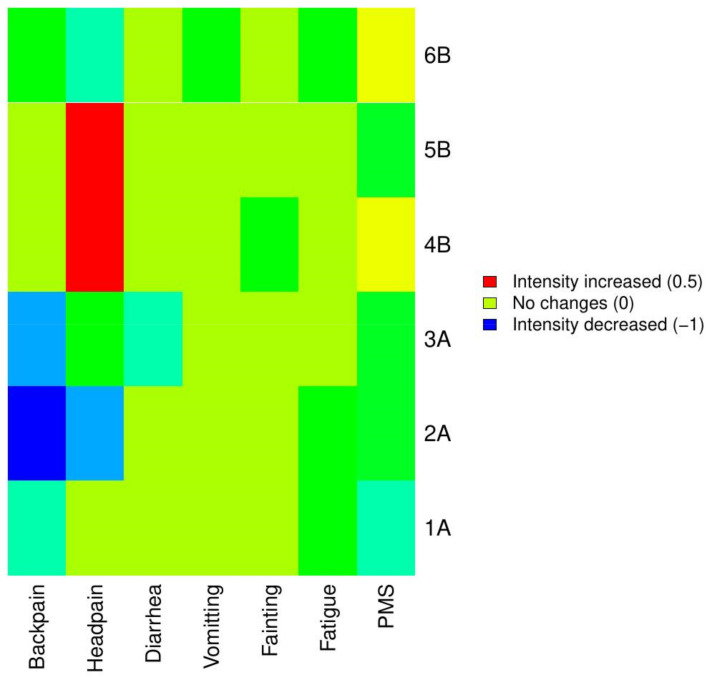
Changes in the intensity of additional symptoms and PMS after treatment among patients with dysmenorrhea in subgroups A and B. Red shows that intensity of symptoms was increased after treatment, whereas blue shows that intensity of symptoms was decreased after treatment. The predominant color in the diagram is that which describes no change in the pain intensity.

The results of our study showed that the treatment was more effective than the use of ibuprofen in reducing the severity of coexisting symptoms in young women with dysmenorrhea. In subgroup A, manual therapy led to a reduction in the severity of back pain in all three patients, while, in subgroup B a similar effect was obtained in only one patient. Manual therapy reduced the intensity of headache in two patients from subgroup A and in one patient from subgroup B. Moreover, in group B, an increase in the severity of headache was demonstrated in two patients while taking ibuprofen. Initially, diarrhea occurred in only one patient from subgroup A and in two women from subgroup B. Manual therapy reduced the severity of diarrhea; this effect was not reported in women taking ibuprofen. Vomiting occurred only in two patients (A3 and B3). In patient A3, manual therapy did not have an impact on the severity of vomiting, while, in patient B3, ibuprofen reduced the condition. There were no episodes of fainting in women from subgroup A, while, in subgroup B, a reduction in syncope with ibuprofen was demonstrated in one patient. The feeling of fatigue during menstruation was present in all the subjects (Figure 8). We demonstrated that manual therapy relieved fatigue in two women, while ibuprofen reduced fatigue in one patient. The outcomes are consistent with the results of the study conducted by Zecchillo et al., who showed that osteopathic therapy was more effective in reducing the severity of vomiting, diarrhea, fatigue, breast tenderness, and headache than a simulation of the therapy [41]. The impact of connective tissue manipulation and reflexology on the symptoms coexisting with menstrual pain was also evaluated by Demirturk et al. The authors demonstrated that both therapies, to a similar extent, reduce the severity of symptoms related to dysmenorrhea [22].

Moreover, our studies showed that, in addition to menstrual pain and the related symptoms, manual therapy decreased the severity of PMS in women from subgroup A. In subgroup B, ibuprofen did not demonstrate an effect on the intensity of PMS. However, it is worth noting that the patients from subgroup B took ibuprofen only from the onset of menstruation, with the lack of a real effect of the drug on the PMS symptoms. A reduction in PMS severity after manual therapy is consistent with the outcomes of the study conducted by Hernandez- Reif et al., who effectively treated PMS with a series of massages [48]. The use of osteopathy and manual therapy has already been described by Shermon et al. [33], who presented five models of osteopathic work to be used in the treatment of particular perimenstrual symptoms.

In our study, an analgesic effect was achieved in all patients from subgroup A and in two patients from subgroup B. However, manual therapy was more effective in reducing the severity of symptoms coexisting with menstrual pain and the severity of PMS symptoms. Moreover, manual therapy led to an improvement in the flexibility and muscle tenderness. On the other hand, patients from subgroup B taking ibuprofen showed no improvement in flexibility or reduction in tenderness of the muscles. Furthermore, one patient with dysmenorrhea from subgroup B showed excessive tension of the tensor fasciae latae and hamstring group muscles.

## 5. Study Limitations

The present study has some limitations that we would like to address. First, our study included no untreated control group, and we cannot exclude a placebo effect. Second, the research presented was based a small group of patients. Further cohort and longitudinal studies are being conducted to verify our findings and to garner a deeper understanding.

## 6. Conclusions

We believe that the decrease in soreness and improvement in muscle flexibility in patients from subgroup A resulted in a reduction in menstrual pain. The study results indicate that manual therapy can be effective in relieving menstrual pain. However, the outcomes require confirmation in studies conducted on a larger group of women with dysmenorrhea.

## Figures and Tables

**Table 1:** Characteristics of patients with dysmenorrhea in subgroup A and B.

Subgroup	Age (years)	BMI (kg/m^2^)	Duration of Menstrual Cycle (days)	Duration of Menstruation (days)
A (*n* = 3) manual therapy	22	21	27	5
B (*n* = 3) ibuprofen	21	23	30	6
The data presented are the mean of each group				

**Table 2:** Duration of menstrual cycle and duration of menstruation in patients in subgroups A and B.

Patient	Duration of Menstrual Cycle (days)		Duration of Menstruation (days)	
	Pre	Post	Pre	Post
1A	28	30	6	6
2A	25	25	5	5
3A	28	33	5	5
4B	34	34	7	7
5B	29	29	6	6
6B	28	26	6	6

Abbreviations: A1, A2, A3, B1, B2, B3—patient designation, PMS—premenstrual Syndrome, Pre—result before treatment: Post—result after treatment.

**Table 3:** Intensity of additional symptoms.

Patient	Backpain		Headpain		Diarrhea		Vomiting		Fainting		Fatigue		PMS	
	Pre	Post	Pre	Post	Pre	Post	Pre	Post	Pre	Post	Pre	Post	Pre	Post
1A	4	2	0	0	0	0	0	0	0	0	3	2	8	3
2A	3	2	4	2	0	0	0	0	0	0	4	2	10	3
3A	3	0	1	0	3	1	1	1	0	0	2	2	3	0
4B	0	0	0	2	4	4	0	0	1	0	3	3	6	7
5B	2	2	0	2	0	0	0	0	0	0	2	2	3	0
6B	3	2	2	0	3	3	1	0	0	0	3	2	7	8

Abbreviations: A1, A2, A3, B1, B2, B3—patient designation, PMS—premenstrual Syndrome, Pre—result before treatment: Post—result after treatment.

**Table 4:** Concentration of progesterone and estradiol 17-beta in patients in subgroup A and B.

Patient	Progesterone (ng/mL)		Estradiol 17-Beta (pg/mL)	
	Pre	Post	Pre	Post
1A	0.54	0.31	53	60
2A	0.42	0.38	26	36
3A	0.26	0.22	5	13
4B	0.58	0.33	13	<5
5B	0.21	0.17	50	21
6B	0.14	0.31	18	25

Abbreviations: A—subgroup with manual treatment; B—subgroup with ibuprofen; Pre—hormone concentration before treatment; Post—hormone concentration after treatment.

## Data Availability

The data presented in this study are available on request from the corresponding author.
